# Understanding long-term continence rates after robot-assisted laparoscopic prostatectomy – one-year follow-up on “Cognitive ability as a non-modifiable risk factor for post-prostatectomy urinary incontinence”

**DOI:** 10.3389/fsurg.2022.1055880

**Published:** 2022-11-23

**Authors:** Mathias Reichert, Hannah Maria Ploeger, Annemarie Uhlig, Arne Strauss, Philipp Henniges, Lutz Trojan, Mirjam Naomi Mohr

**Affiliations:** ^1^ Department of Urology, University Medical Center Goettingen, Goettingen, Germany; ^2^ Department of Pediatrics, University Hospital Bonn, Bonn, Germany

**Keywords:** long-term continence rates, cognitive ability, nerve sparing, post-prostatectomy incontinence, predictor, prostate cancer, robot-assisted radical prostatectomy

## Abstract

**Purpose:**

To evaluate long-term continence rates (12 months) in patients after robot-assisted laparoscopic prostatectomy (RALP) in relation to their cognitive ability (CoAb), which proved to be a predictor for early post-prostatectomy incontinence.

**Material & Methods:**

This is the 12-month follow-up evaluation of our previously published observational single-center, prospective evaluation of 84 patients who underwent RALP as treatment of their localized prostate cancer between 07/2020 and 03/2021. Post-prostatectomy incontinence (PPI) was measured by asking patients about their 24 h pad usage, whereby 0 pads were considered continent and ≥1 pad was considered incontinent. CoAb was evaluated by performing the Mini-Mental State Examination prior to surgery. Possible predictors for PPI were evaluated using univariate and multivariable logistic regression models.

**Results:**

Multivariable logistic regression analyses identified early incontinence status and nerve sparing (NS) as independent predictors for PPI after 12 months, resulting in a 5.69 times higher risk for PPI when the loss of urine was between 10 and 50 ml during the early performed pad test (one day after catheter removal) compared to 0–1 ml loss of urine [95% confidence interval (CI): 1.33–28.30, *p* = 0.024] and a 6.77 times higher risk for PPI, respectively, when only unilateral NS was performed compared to bilateral NS (95% CI: 1.79–30.89, *p* = 0.007). CoAb lost its predictive value for long-term PPI (*p* = 0.44).

**Conclusion:**

The results of this study suggest that PPI is a dynamic, rather than a static condition with a dynamically changing pathophysiology within the first 12 months after RALP. Coping methods and therapies should adapt to this circumstance.

## Introduction

Post-prostatectomy incontinence (PPI) is a severe and frustrating condition which impairs patients' quality of life and has an enormous impact on the overall socioeconomic burden of diseases.

The entire etiology of PPI is not entirely understood thus far (1). Focus had been laid on different possible pathophysiologic factors, such as damage to the striated muscle (2, 3), urodynamic changes (4), or damage to the nervous supply (3), alongside patients' age at time of surgery (3, 5), a shorter (preoperative) membranous urethral length (6) or bigger prostate gland volume (3, 7).

In 2021 we demonstrated in our study that cognitive ability (CoAb) seems to have an influence on early post-prostatectomy incontinence (ePPI) (8). When classified in modifiable and non-modifiable risk factors, we concluded, that the CoAb should be classified as a non-modifiable risk factor for ePPI. In our opinion, this is a very interesting issue since up until then, focus had never been laid on this topic before. However, since cognitive decline seems to have a negative influence on the development and the course of diseases (9, 10), why not on the course of PPI?

Interestingly, PPI after robot-assisted laparoscopic prostatectomy (RALP) can regress – or continence can be improved – within the first 12–24 months after radical prostatectomy (RPE), but the main improvement seems to be observed within the first year (11).

Although the International Consultation on Incontinence-Research Society recommends pelvic floor muscle exercise (PFME)/ pelvic floor muscle training (PFMT) prior to RPE to prevent PPI or shorten the time of continence recovery postoperatively (12) and guidelines recommend their use (13), the influence of PFME/PFMT (pre- and postoperatively) on the convalescence after RPE remains uncertain (8).

It has been shown that contracting the pelvic floor seems to be difficult for some people, especially male patients (14) and can lead to insufficient PFME/PFMT. In order to perform sufficient PFME/PFMT, such as the “Kegel-Maneuvers” (15), there has to be certain body awareness and understanding.

As mentioned in our study about the influence of CoAb on ePPI (8), the 12-month follow-up of the trial should greatly further our understanding of the influence CoAb has on the emergence of PPI or on the recovery of continence.

## Material and methods

This study is the 12-month follow-up evaluation of our previously published study “Cognitive ability as a non-modifiable risk factor for post-prostatectomy urinary incontinence: a double-blinded, prospective, single-center trial” (8).

The data has been collected prospectively. The study-population consists of patients who underwent robot-assisted laparoscopic prostatectomy (RALP) in the department of urology at the University Medical Center Goettingen between 07/2020 and 03/2021. The institutional review board of the University Medical Center Goettingen approved this study.

Indications and exclusion criteria were described as followed (8): The indication for RALP was localized prostate cancer (PCa) detected by biopsy. All patients were staged according to current guidelines (German S3 Guidelines, EAU Guidelines, respectively) (16, 17).

Exclusion criteria were poor patient literacy, which could lead to incorrect, bad Mini-Mental State Examination (MMSE) results, and an interdisciplinary board decision to initiate a multimodal therapy before surgery.

The aim of this follow-up was – after assessing the interrelation between MMSE and PPI in the very early phase (one day after catheter removal 5–7 days post-surgery) and showing that there is a significant correlation between a bad MMSE result and PPI occurrence (8) – to evaluate the long-term influence of a bad MMSE result on PPI (after 12 months).

The perioperative standard was described as followed by Reichert et al. (8): All patients were seen at least one day before surgery. After study inclusion they underwent the MMSE by Folstein et al. (18), always carried out by the same physician to prevent unequal distortion of the data. Surgeons and patients had no information about the test result (double-blinded). While 30 points are always the maximum a patient can achieve, the further MMSE subdivisions are defined inconsistently in literature. In this study, the cut-offs were defined based on clinical performance in the MMSE and the median (28 ± 1.6), yielding the following subdivisions: 28–30 points = no cognitive deficit, 25–27 points = mild cognitive deficit, and ≤24 points = severe cognitive deficit.

During the preoperative assessment, patients were asked if they had performed pelvic floor training, instructed by physiotherapists, before surgery.

All RALPs were performed by one of three surgeons using the DaVinci SI system. All surgeons had an experience of at least 450 RALPs, each. The patient allocation was randomized. The surgical techniques, e.g., preservation and reconstruction of the pelvic floor, were standardized (e.g., Rocco Stitch, etc.) (19). Preservation of the neurovascular bundle (NVB) was performed whenever the oncological option with respect to the guidelines and the intraoperative findings were given and the patient asked for it. Preoperative erectile function was evaluated by the standardized IIEF questionnaire. For oncological safety we performed a frozen section of the entire lateral part of the gland surfacing the NVB (from urethra to the bladder neck) during the RALP. When there was a cancer-positive area of the margin, the corresponding bundle was resected. Nerve sparing (NS) was assigned categorically into “no NS,” “unilateral NS” and “bilateral NS.” A transurethral catheter was placed in all patients after surgery. No suprapubic catheters were used.

The postoperative course applied to the entire patient collective was standardized with regard to the use of analgesics, diet, and physiotherapy. The decision about the duration of the transurethral catheter placement was dictated by the surgeons on basis of the intraoperative course (planned for 5 or 7 days). Before removing the tube, a radiological control (cystogram/retrograde urethrocystography) was performed. If there was no leakage of the contrast medium at the vesicourethral anastomosis, the catheter was removed accompanied by standardized instructions by a physician to train the pelvic floor.

Patients stayed hospitalized for at least 24 h after catheter removal and received standardized pelvic floor training by physiotherapists within 5 h after catheter removal. The patients documented their voiding conditions using a standardized micturition protocol for 24 h (voiding rates, amount per fraction, pad usage, etc.). The next day urinary incontinence/continence was examined again by the standardized 1 h pad test by the International Continence Society (20). Before discharge, ultrasonography of post voiding residual urine volume was performed.

MMSE results were also categorized into “good” (no cognitive deficit), “intermediate” (mild cognitive deficit) and “bad” (severe cognitive deficit).

For the 12-month follow-up, patients were contacted and asked about their pad usage. 0 pads were considered a good long-term continence status, ≥1 pad per day a bad long-term continence status.

### Statistical analysis

Categorical variables were described with absolute number and corresponding percentage; continuous variables were described using mean with standard deviation (SD), and median with range. Statistical comparisons of categorical variables between groups were performed using the Chi square test. Continuous variables were compared using the Wilcoxon rank sum test or Student´s t-test based on evaluation of normal distribution by the Shapiro Wilks test (21). Binary univariate and multivariable logistic regression was used for identification of predictors. Variables were considered for inclusion in multivariable models accordingly to their literature-based influence on the outcome and based on statistical significance (*p* < 0.1) from univariate logistic regression analysis and retained in the final multivariable model if *p* < 0.05. The final multivariable logistic regression models were assessed for goodness-of-fit (calibration) with the Hosmer-Lemeshow test (22), and for discrimination with the Area Under the Curve (AUC) statistic. All statistical analyses were performed with R version 4.1.0 (R Core Development Team, Vienna, Austria) and RStudio version 1.3.1093 (RStudio Inc., Boston, MA). Statistical tests with *p* < 0.05 were considered significant. All *p*-values are two-sided.

## Results

84 patients showed a complete record of their data, including a complete MMSE result and a complete postoperative follow-up with a complete pad test in the very early phase (one day after catheter removal), a sufficient micturition protocol, and a 12-month follow-up with a report of their pad usage.

After 12 months of surgery following distributions of the 24 h pad usage can be seen (Table 1).

**Table 1:** Descriptive analysis of the total number of pad usage after 12 months.

Number of pads	Patients (*n *= 84)	Percentage (%)
0	50	59.5
1	26	31.0
2	8	9.5

We categorized patients with 0 pads per day as “continent” and ≥1 pad per day as “incontinent” (after 12 months). Patients characteristics in both groups are shown in Table 2, including their histopathological findings in the prostatectomy specimen.

**Table 2:** Patient characteristics between the “continent” and the “incontinent” group.

		Total (*n* = 84)	Continent (*n* = 50)	Incontinent (*n* = 34)	*p*-value
Age (years)	Median (min; max)	64 (48;78)	64 (48;78)	65 (58;78)	0.22
BMI (kg/m²)	BMI <24	9 (10.7 %)	6 (66.7 %)	3 (33.3 %)	0.78
	BMI 24–<30	58 (69.0 %)	35 (60.3 %)	23 (39.7 %)	
	BMI 30–<35	14 (16.7 %)	8 (57.1 %)	6 (42.9 %)	
	BMI ≥35	3 (3.6 %)	1 (33.3 %)	2 (66.7 %)	
iPSA (ng/ml)	<4	4 (4.8 %)	3 (75.0 %)	1 (25.0 %)	0.12
	4–<10	51 (60.7 %)	30 (58.8 %)	21 (41.2 %)	
	10–<20	17 (20.2 %)	13 (76.5 %)	4 (23.5 %)	
	≥20	12 (14.3 %)	4 (33.3 %)	8 (66.7 %)	
Prostate volume (ml)	<40	39 (46.4 %)	18 (46.2 %)	21 (53.8 %)	0.05
	40–90	43 (51.2 %)	30 (69.8 %)	13 (30.2 %)	
	>90	2 (2.4 %)	2 (100 %)	0	
IPSS (preoperative)	<8	47 (57.3 %)	32 (68.1 %)	15 (31.9 %)	0.16
	8–19	31 (37.8 %)	17 (54.8 %)	14 (45.2 %)	
	20–35	4 (4.9 %)	1 (25.0 %)	3 (75.0 %)	
ICIQ (preoperative)	No incon.	58 (75.3 %)	35 (60.3 %)	23 (39.7 %)	0.45
	Light incon.	14 (18.2 %)	8 (57.1 %)	6 (42.9 %)	
	Mid incon.	1 (1.3 %)	1 (100 %)	0	
	Severe incon.	4 (5.2 %)	1 (25.0 %)	3 (75.0 %)	
pT status	pT2	14 (16.7 %)	9 (64.3 %)	5 (35.7 %)	0.92
	pT3-4	70 (83.3 %)	41 (58.6 %)	29 (41.4 %)	
pN status	pN0	78 (94.0 %)	48 (61.5 %)	30 (38.5 %)	0.17
	pN1	5 (6.0 %)	1 (20.0 %)	4 (80.0 %)	
R status	R0	61 (72.6 %)	39 (63.9 %)	22 (36.1 %)	0.25
	R1	22 (26.2 %)	11 (50.0 %)	11 (50.0 %)	
	R2	1 (1.2 %)	0	1 (100 %)	
GS	6	1 (1.2 %)	0	1 (100 %)	0.03
	7	65 (77.4 %)	43 (66.2 %)	22 (33.8 %)	
	8	4 (4.8 %)	0	4 (100 %)	
	9	14 (16.7 %)	7 (50.0 %)	7 (50.0 %)	
NS	Unilateral NS	27 (32.1 %)	11 (40.7 %)	16 (59.3 %)	0.01
	Bilateral NS	25 (29.8 %)	21 (84.0 %)	4 (16.0 %)	
	No NS	32 (38.1 %)	18 (56.2 %)	14 (43.8 %)	
Pelvic floor training preoperative	Yes	13 (23.6 %)	9 (69.2 %)	4 (30.8 %)	0.30
	No	42 (76.4 %)	20 (47.6 %)	22 (52.4 %)	
MMSE	Good	53 (65.4 %)	31 (58.5 %)	22 (41.5 %)	0.44
	Intermediate	26 (32.1 %)	14 (53.8 %)	12 (46.2 %)	
	Bad	2 (2.5 %)	2 (100 %)	0	
Catheterization time (after RALP)	≤7 days	78 (92.9 %)	47 (60.3 %)	31 (39.7 %)	0.95
	≥8 days	6 (7.1 %)	3 (50.0 %)	3 (50.0 %)	

(min, minimum; max, maximum; BMI, Body Mass Index; iPSA, initial prostate specific antigen; ng/ml, nanogram per milliliter; ml, milliliter; IPSS, international prostate symptom score; ICIQ, international consultation of continence questionnaire; incon., incontinence; GS, Gleason Score; NS, nerve sparing; MMSE, Mini-Mental State Examination; RALP, robot-assisted laparoscopic prostatectomy).

Patients who had micturition issues preoperatively with a higher IPSS score and a worse ICIQ score showed no significant higher pad usage per day 12 months postoperatively (*p* = 0.16, *p* = 0.45 respectively). This is similar to the findings for ePPI (*p* = 0.11 (IPSS), *p* = 0.77 (ICIQ)) (8).

Regarding age at time of surgery (*p* = 0.22) and BMI (*p* = 0.78), significance was not reached, as in our previously published data (*p* = 0.11, *p* = 0.55) (8). The iPSA had been significant in the univariate analysis the day after catheter removal (*p* = 0.05) (8) but lost its significance in the follow-up (*p* = 0.12).

Histopathologic characteristics of both groups (continence vs. incontinence) were comparable in regard of T status (*p* = 0.92) and N status (*p* = 0.17) but showed a significant difference in GS (*p* = 0.03). The resection status was not significant for ePPI as well as for the 12-month follow-up (*p* = 0.46, *p* = 0.25 respectively) (8).

In univariate analyses, a higher GS and a larger prostate volume reached significance status for long-term PPI (*p* = 0.03, *p* = 0.05 respectively).

PFME/PFMT performed preoperatively showed no significant influence on continence status in the early phase (*p* = 0.54) (8) and stayed insignificant 12 months after (*p* = 0.30).

Patients with an insufficient cystogram 7 days post-surgery had all shown a loss of ≥2 ml urine in the early pad test one day after catheter removal and therefore had been considered incontinent in the early phase (*p* = 0.06) (8). The number of these patients is too small for further analysis in the 12-month follow-up and therefore no statement about the influence on long-term continence can be made.

In the univariate analysis in the early phase after catheter removal, MMSE showed a significant correlation between CoAb and ePPI (Table 3) (8).

**Table 3:** Significant correlation between MMSE results and continence status, whereby dry is defined as <2 ml loss of urine and wet ≥2 ml during the 1 h pad test one day after catheter removal (8).

		Total	Dry	Wet	*p*-value
MMSE	Good	70 (66.7 %)	34 (48.6 %)	36 (51.4 %)	0.01
	Intermediate	33 (31.4 %)	7 (21.2 %)	26 (78.8 %)	
	Bad	2 (1.9 %)	2 (100 %)	0	

MMSE, Mini-Mental State Examination.

MMSE was considered an independent predictor of ePPI in univariate and multivariable logistic regression analyses after adjustment for NS (Table 4).

**Table 4:** Univariate and multivariable logistic regression regarding ePPI, one day after catheter removal (8).

	Outcome	ORunivariate	ORmultivariable
MMSE	Good (reference)	1	−
	Intermediate	3.51 (1.40–9.73, *p* = 0.010)	3.17 (1.22–9.06, *p* = 0.023
	Bad	No OR calculated	No OR calculated
NS (binary)	NS (reference)	1	−
	No NS	3.53 (1.51–8.89, *p* = 0.005)	3.93 (1.54–11.09, *p* = 0.006)

ePPI, early post-prostatectomy incontinence; OR, Odds Ratio; MMSE, Mini-Mental State Examination; NS, nerve sparing.

But in the 12-month follow-up, MMSE lost its significant status (*p* = 0.44) (Table 1).

Univariate and multivariable logistic regression analyses identified the loss of urine in the pad test on the day after catheter removal and NS as independent predictors for long-term PPI (after 12 months) (Table 5).

**Table 5:** Univariate and multivariable logistic regression regarding long-term PPI (12 months).

	Outcome	ORunivariate	ORmultivariable
Pad test ePPI	Good continence (reference)	1	−
	Light incon.	3.62 (1.25–11.30, *p* = 0.021)	3.82 (1.21–13.23, *p* = 0.026)
	Intermediate incon.	6.94 (1.84–29.67, *p* = 0.006)	5.69 (1.33–28.30, *p* = 0.024)
	Severe incon.	5.79 (0.81–51.04, *p* = 0.081)	6.65 (0.83–66.00, *p* = 0.078)
NS (binary)	NS bilateral (reference)	1	−
	NS unilateral	7.64 (2.20–32.04, *p* = 0.002)	6.77 (1.79–30.89, *p* = 0.007)
	No NS	4.08 (1.22–16.47, *p* = 0.031)	2.48 (0.64–10.97, *p* = 0.203)

PPI, post-prostatectomy incontinence, OR, Odds Ratio; ePPI, early post-prostatectomy incontinence; incon., incontinence; NS, nerve sparing.

Upon multivariable analyses, patients with an intermediate ePPI have a 5.69 times higher risk of long-term PPI when compared to patients with a good early continence status [95% confidence interval (CI): 1.33–28.30, *p* = 0.024], after adjustment for NS (Table 5). We considered 0–1 ml in the early 1 h pad test as a good continence status, 1–10 ml as a light, 10–50 ml as an intermediate and >50 ml as a severe incontinence status, respectively.

Likewise, patients who did not receive a NS have a 6.77 times higher risk of PPI (95% CI: 1.79–30.89, *p* = 0.007).

Model diagnostics revealed adequate model calibration and acceptable discrimination (AUC = 0.783).

## Discussion

Continence recovery after RALP seems to be possible up to 24 months, although the main improvement takes place during the first 12 months (11). Several studies showed different predictors for PPI (2, 3, 5, 8) and different circumstances leading to a better recovery and to a good continence status after RALP, like pelvic floor integrity, including neurovascular integrity (23).

The importance of the neurovascular supply was topic in the review by Reeves et al. 2015 (24). They showed that the preservation of the NVB was associated with a shorter time of recovery to a good postoperative continence level with continence rates of 42.2% (at six weeks), 64.8% (at three months), 88.9% (at six months) and 83.9% (at 12 months), respectively (24). Steineck et al. postulated that bilateral NS is better than unilateral NS in regards to PPI 12 months after surgery (25). They found a 2.37 times higher risk of incontinence when no NS was performed, and a 1.78 times higher risk when only one NVB could be preserved at the end of surgery. Limitations of their study included the unstandardized surgical steps within a multicenter study. Our results support their conclusion, since there was a 6.77 times higher risk of developing a PPI in a one-year follow-up, when only unilateral preservation of the NVB was reached, compared to a bilateral NS (95% CI: 1.79–30.89, *p* = 0.007).

The development and detailed pathophysiology of urinary incontinence is not entirely understood and remains unclear (1). Risk factors leading to PPI can be distributed in patient-sited and surgery-sited risk factors. Surgery-sited risk factors seem obvious, like damaging the external striated sphincter, or disintegrating the neurovascular supply (24, 25). Patient-sited risk factors include anatomical prerequisites that have a higher risk leading to PPI, like older age at time of surgery (3, 5), bigger prostate gland volume (3, 7), or a shorter (preoperative) membranous urethral length (6). Preoperative micturition issues, like obstructive micturition issue (IPSS) or incontinence status (ICIQ) proved to have a predictive value in other studies (26). In our 12-month evaluation there was no significant difference between the continent and incontinent group.

In our previously published study “Cognitive ability as a non-modifiable risk factor for post-prostatectomy urinary incontinence” (8) we could show in multivariate logistic regression analyses the important influence of CoAb on ePPI.

In the very early phase after catheter removal after RALP, patients with an intermediate MMSE result (25–27 points) had a 3.17 times higher risk of ePPI when compared to patients with a good MMSE result (≥28 points) (95% CI: 1.22–9.06, *p* = 0.023). A reason for this might be the inability of controlling the pelvic floor sufficiently (8).

We concluded that CoAb (confirmed by MMSE) as a surrogate parameter for pelvic floor contraction should be treated as a non-modifiable risk factor for ePPI and as such should lead to a better preparation of the patient before surgery (8). Before we published our findings, it has already been discussed that patients with non-modifiable risk factors should be offered more intense targeted preoperative physiotherapy interventions (27).

It was postulated that a bad MMSE result should not lead to a change in the decision regarding the chosen therapy, since RPE is the only therapy that provides a better oncological outcome of the localized disease than watchful waiting (28).

Interestingly, in this follow-up study, CoAb could not function as a predictor for PPI anymore, apart from the predictive value of ePPI on PPI after 12 months, as the MMSE results lost their significance. In our opinion these findings only support the statement, that CoAb should not influence the decision making on curative therapy.

Voluntary micturition in a non-operated patient happens subconsciously but requires highly complex nervous supply and interaction without disturbance. The same complex interaction is needed to enable continence. After RALP, the physiologic conditions changed extremely, and the subconscious interaction with the pelvic floor has to be transferred into conscious interaction. This process requires a high level of willingness, coordination etc. in the patient.

From the day of catheter removal after RPE, patients must be able to compensate the missing effect of the prostate on continence since the gland was removed. Lower CoAb could lead to an insufficient conscious pelvic floor contraction in the early phase after RPE and therefore lead to a higher rate of ePPI.

This effect seems to lose its significant impact on PPI within the first year after RALP since patients with lower CoAb reach similar continence status compared to patients with higher CoAb. A reason explaining the lack of significance of CoAb on PPI could be, that patients with lower CoAb need more time to learn using their pelvic floor sufficiently.

Nevertheless, these results emphasize the importance of a better targeted preparation of the patients with a worse MMSE result, since a better early recovery could be reached within this year.

Sayner and Nahon (2020) postulated that optimal postoperative urinary continence outcomes could be reached by an individualized prescription of PFME/PFMT (29). The exercises should be taught in such a way that every individual patient can follow. In the early phase, patients with a lower CoAb could have more problems to consciously contract the pelvic floor so that the required mid-urethral occlusion pressure can be reached.

Performing PMFE the right way seems to be the key for developing greater pelvic muscle strength. This was confirmed by a review done by Jacomo et al. in which they concluded that Pilates, the Paula method, and hypopressive exercises performed alone do not increase pelvic floor muscle strength, but PFMT continues to be the gold standard (30).

In our study, preoperatively performed PFME had no influence on PPI, neither on ePPI nor on PPI after 12 months. This is comparable to existing studies so far. Geraerts et al. compared the influence of preoperative and postoperative PFMT with only postoperative PFMT and saw no significant difference in the 24 h pad test. The median time to continence in both groups was 30 and 31 days (31).

We believe preoperatively performed PFME does not protect continence postoperatively since the whole changes occurring during RALP provide a completely new pelvic floor anatomy not comparable with the preoperative status.

Concluding the results of this study, NS still has a predictive value for long-term continence rates. As mentioned above, some researchers suggest that NS shortens the time of continence recovery (24). Maybe in our study, the recovery to the maximum continence status was not reached by every patient within the first 12 months.

Another significant parameter after 12 months was gland volume and GS. In our previously published results, none of them reached significant status in the early phase after RALP (8).

Our study shows that there is a shift of significant parameters for PPI within one year after RALP, since MMSE lost its predictive value for PPI for example and others reach significant status. This shift suggests that the pathophysiology of PPI after RALP changes. Remarkably, patient-sited risk factors like MMSE lose their predictive value, but on the other hand surgical-sited risk factors, like NS, prove to be still predictive for PPI.

For us this is a confirmation that the pathophysiology of PPI in combination with the patientś compensation mechanisms change dramatically within the first year after RALP. This is a very important step in the understanding of PPI after RALP.

At the beginning, conscious, controllable coping mechanisms must be strengthened and intensified, but as the process progresses, their relevance gradually recede into the background. The surgical related reasons move into the foreground as time progresses.

This knowledge can be the next step in understanding PPI and its pathophysiology and can help providing better care for patients suffering from it.

This study has some limitations worth mentioning. Continence status develops to its best during the first 24 months after RALP (11). Our long-term follow-up lasts 12 months. Most of the improvement happens during the first 12 months, but we cannot predict the outcome after 24 months. A follow-up after 24 months could confirm the changing evolvement of PPI after RALP. Furthermore, we cannot postulate when the switch between the significant predictors happens. When does the intense focus on postoperative PFMT and individualized training stop being so important? To answer this question a more detailed follow-up after 3 months, 6 months and 9 months would be desirable.

A bad MMSE result – and with it lower CoAb – is seen as a surrogate parameter for a worse body awareness and leads to an insufficient performance of PFMT/PFME. This argument should be proven with a direct examination of MMSE results and performing pelvic floor muscle contraction measured *via* pelvic floor electromyography.

We included 84 patients into this follow-up study. A bigger patient population, maybe in a multi-center study, could strengthen our argumentation.

The measurement of continence status was done asking the patients about their pad usage per day. Recent studies suggested that PROMS, like EPIC-26, should be performed instead of just considering the pad usage (32). But to do so, the preoperative EPIC-26 questionnaire must be considered.

## Conclusion

The results of this study, a follow-up of our previously published study about CoAb as a non-modifiable risk factor for PPI, suggest that PPI is a dynamic, rather than a static condition. Lower CoAb, which is significantly associated with a higher incidence of ePPI, loses its predictive value for PPI. However, the significance of surgical-sited risk factors on the development of PPI seems to increase. The pathophysiology of PPI dynamically changes over 12 months after RALP. Coping methods and therapies should adapt to these circumstances.

## Data Availability

The data that support the findings of this study are available from the corresponding author upon reasonable request.
